# Effects of a Functional Cone Mushroom (*Termitomyces fuliginosus*) Protein Snack Bar on Cognitive Function in Middle Age: A Randomized Double-Blind Placebo-Controlled Trial

**DOI:** 10.3390/nu16213616

**Published:** 2024-10-24

**Authors:** Supaporn Muchimapura, Wipawee Thukham-mee, Terdthai Tong-un, Weerapon Sangartit, Sophida Phuthong

**Affiliations:** 1 Department of Physiology, Faculty of Medicine, Khon Kaen University, Khon Kaen 40002, Thailand; supmuc@kku.ac.th (S.M.); meewep@gmail.com (W.T.-m.); terdthai@kku.ac.th (T.T.-u.); weerasan@kku.ac.th (W.S.); 2 Human High Performance and Health Promotion Research Institute, Khon Kaen University, Khon Kaen 40002, Thailand

**Keywords:** cone mushroom, cognitive function, memory, functional snack bar

## Abstract

**Background**: Due to the rising prevalence of cognitive impairment in the middle-aged and elderly population, combined with consumer demand for functional foods to improve health and well-being. **Objective**: This study aimed to formulate a functional cone mushroom (*Termitomyces fuliginosus*) (FCM) protein snack bar and evaluate its amino acid profile, phytochemical contents, biological activity and impact on cognitive function. **Methods**: A total of 26 middle-aged male and female participants were randomized and divided into placebo, FCM1 and FCM2 groups. Continuous consumption was performed for 6 weeks. Demographic data, body composition, cognitive function and memory were evaluated at baseline and at the end of the study period (6 weeks). **Results**: The event-related potential (ERP) analysis results showed a significant increase in N100 and P300 amplitude at the Fz location in participants who consumed the functional cone mushroom protein snack bar at a dose of 1 g compared to the placebo group (*p* = 0.015). Additionally, subjects who consumed the functional cone mushroom protein snack bar at a dose of 2 g showed a significantly increased P300 amplitude and percent accuracy of numeric working memory (*p* = 0.048) compared to those in the placebo group (*p* = 0.044). The possible underlying mechanism may involve AChE and MAO suppression activity alongside antioxidant activity. **Conclusions**: These data suggest that FCM can improve cognitive function and memory and may be considered for use in natural supplementation products with possible health benefits.

## 1. Introduction

Impaired cognitive function is characterized by loss of memory, learning difficulties and decreased attention, leading to a decrease in life quality and an increase in the risk of dementia and mortality [1]. Epidemiological studies report a rising prevalence of cognitive and memory impairment in the general population worldwide, particularly among the middle-aged and elderly groups [1,2]. Preventive interventions for at-risk population subgroups should be sought to prevent cognitive impairment and maintain functional ability, promoting overall well-being. Several studies have demonstrated that cognitive function can be maintained, or even improved, through the regular consumption of healthy foods, and sufficient supplementation with functional ingredients enhances brain health and performance [3,4,5,6].

Given the growing market for ready-to-eat products, snack bars are considered an appropriate option for consumers’ lifestyles [7]. This formulation offers several benefits, including convenient storage, high nutritional content and ease of consumption [8]. However, the customer also prefers to consume a convenient product that has some benefit to their health [9]. Therefore, the formulation of innovative functional food products containing functional ingredients is projected to align with consumer needs and be beneficial for maintaining and improving cognitive processes.

The cone mushroom (*Termitomyces fuliginosus*) is widely found in Thailand and other Asian countries [10]. It is rich in protein, dietary fiber and several bioactive compounds and possesses various beneficial properties, including anticancer, antimicrobial and antioxidant effects [10,11,12,13]. It has been revealed that several types of mushrooms may potentially impact neuroprotective and neuro-regenerative properties [10,14,15,16,17]. Previous studies have suggested the association between edible mushroom intake and cognitive function [10,18]. Mushroom supplementation has been associated with improved mood and memory, alongside increased circulating pro-brain-derived neurotrophic factor (BDNF) and cognitive-related neurotransmitters [14,19]. Cognitive impairment has been found to be associated with a decline in neurotransmitter levels, especially acetylcholine (ACh) and dopamine [20,21,22], while supplementation with mushrooms has restored the levels of these neurotransmitters [19,23]. Several bioactive compounds have been observed in termite mushrooms, which could explain their effects on improving age-related neurodegenerative disorders. These compounds include polysaccharides, dietary fiber, flavonoids and several types of phenolic compounds, such as phenolic acid, gallic acid and quercetin [10,24]. Among them, the flavonoid metabolites easily pass the blood–brain barrier and have shown potential for improving cognitive function and memory via their antioxidant activity [25,26]. Flavonoids are powerful antioxidants that help combat oxidative stress by scavenging free radicals, which is crucial for brain health, as excessive oxidative stress is associated with cognitive decline and neurodegenerative diseases [27,28]. By protecting neuronal cells from oxidative damage, flavonoids support overall cognitive health [29,30].

However, few studies have examined the functional components of *Termitomyces fuliginosus* and their impact on cognitive function. Thus, this study aims to formulate a functional cone mushroom protein snack bar and evaluate its amino acid profile, phytochemical contents, and biological activity. Additionally, we aim to evaluate its impact on cognitive function and memory in middle-aged volunteers through a randomized double-blind placebo-controlled trial study.

## 2. Materials and Methods

### 2.1. Preparation of a Functional Cone Mushroom Protein Snack Bar

#### 2.1.1. Preparation of a Cone-Mushroom-Derived Protein Concentrate

Functional protein was extracted by the method previously described [26]. Extraction was performed under alkaline conditions utilizing 2 N NaOH and agitation at 1000 rpm for 2 h. The solution was subsequently centrifuged at 8000× *g* for 20 min at 4 °C. The supernatant was gathered and adjusted to pH 4.5 to precipitate the protein. A concentrated protein pellet was obtained after stirring the solution at 1000 rpm for 1 h, followed by centrifugation at 8000× *g* for 20 min at 4 °C. This concentrated protein pellet was then mixed with distilled water in a ratio of 1:10, and the solution’s pH was adjusted to 7.0. Stirring at 1000 rpm for one hour yielded a concentrated protein solution. The solution underwent additional centrifugation at 8000× *g* for 20 min, followed by the incorporation of 5% maltodextrin to achieve a homogenous mixture, preparing it for the subsequent spray-drying procedure.

#### 2.1.2. Preparation of a Functional Cone Mushroom Protein Snack Bar

The process was divided into 7 steps: Step (1) Whole grains, such as almonds, watermelon seeds, pumpkin seeds and cashew nuts, were roasted in an oven at 120 °C for 15-20 min (until they turned light brown and smelled appealing) and then left to cool. Step (2) Sticky ingredients such as dried dates, raisins and dried bananas were blended. Step (3) Dry powdered ingredients, including roasted coconut husks, cocoa powder and shiitake mushroom powder, were mixed together. (4) The ingredients from steps 2 and 3 were combined until smooth. Step (5) The ingredients from step 4 were mixed with the prepared toasted grains (almonds, watermelon seeds, pumpkin seeds and cashew nuts), mixing in the last step to reduce the breakage of the grains. Step (6) The mixture was divided into 20 g pieces and then pressed into a mold or pressed into a rectangular shape (approximately 3 × 7 cm, 1 cm thick). Step (7) The pieces were baked in a hot air oven at 60 degrees Celsius for 90 min and then left to cool and packaged.

### 2.2. Determination of Total Phenolic Content of a Function Cone Mushroom Protein Snack Bar

Total phenolics were evaluated by the Folin–Ciocalteu method. In brief, we prepared a 96-well plate for mixing the Folin–Ciocalteu reagent with the homogenate function cone mushroom protein snack bar. After 8 min of incubation, Na_2_CO_3_ solution was added, and the mixture was further incubated in dark conditions at room temperature for 2 h. The absorbance was measured at 765 nm. The total phenolic content is expressed as the gallic acid equivalent (GAE), in milligrams of gallic acid equivalent (GAE)/g of sample [26,31].

### 2.3. Determination of Total Flavonoid Content in a Functional Cone Mushroom Protein Snack Bar

The total flavonoids were quantified using a colorimetric method according to the method previously described [26]. The homogenate function cone mushroom protein snack bar was mixed with 2% aluminum chloride and then incubated for 1 h at room temperature. The absorbance of the mixtures was quantified using a microplate reader relative to a blank at 415 nm. Quercetin served as the reference standard. The results are shown as milligrams of quercetin equivalent (QE) per gram of snack bar sample.

### 2.4. Determination of the Amino Acid Profile of a Cone-Mushroom-Derived Protein Concentrate or Functional Cone Mushroom (FCM) Protein Snack Bar

One hundred grams of a protein concentrate derived from the cone mushroom, or a functional cone mushroom (FCM) snack bar, was sent to the Central Laboratory (Khon Kaen, Thailand) Co., Ltd., for analysis and reporting of the amino acid profile using high-performance liquid chromatography (HPLC).

### 2.5. Determination of Antioxidant Activity

#### 2.5.1. DPPH (1,1-Diphenyl-2-Picrylhydrazyl Radical) Inhibition of a Functional Cone Mushroom (FCM) Snack Bar

In brief, DPPH solution was aliquoted in methanol and mixed with 20 μL of the sample at various concentrations at 5 to 1000 μg/mL, then incubated for 30 min at room temperature with dark conditions. After incubation, the sample was measured with a microplate reader at an absorbance of 517 nm. The results are expressed as the half-maximal inhibitory concentration (IC50) in mg per mL and the percentage of inhibition of DPPH radical formation, respectively [26].

#### 2.5.2. ABTS (2,2′-Azino-Bis-(3-Ethylbenzthiazoline-6-Sulphonic Acid Radical) Inhibition of a Functional Cone Mushroom (FCM) Snack Bar

The ABTS radical scavenging method was carried out as previously published for a protein extract from cone mushroom [26]. In summary, A 20 μL aliquot of the snack bar sample at different concentrations was combined with 40 μL of distilled water and 150 μL of ABTS solution, and the absorbance at 734 nm was measured using a microplate reader. The Trolox solution served as the standard. The findings are expressed as the IC50 value and the percentage of suppression of ABTS radical production, respectively.

### 2.6. Determination of Anti-Inflammation Activities by COX-II (Cyclooxygenase-II) Inhibition of a Functional Cone Mushroom (FCM) Snack Bar

COX-II activity was assessed using a colorimetric approach in accordance with the assay kit manufacturer’s protocol (Cayman Chemical, Ann Arbor, MI, USA) [32]. A reaction mixture comprising 150 μL of assay buffer, 10 μL of cone mushroom protein extracts, 10 μL of heme, 10 μL of COX-II working solution, 20 μL of 10 μM TMPD (*N*,*N*,*N*′,*N*′-tetramethyl-p-phenylenediamine dihydrochloride) (Sigma-Aldrich, Saint Louis, MO, USA) and 20 μL of 100 μM arachidonic acid was introduced into 96-well microtiter plates and incubated at ambient temperature for 30 min. Subsequently, the microtiter plates were assessed for absorbance at 590 nm. The findings are expressed as the IC50 value. Indomethacin served as the reference compound.

### 2.7. Determination of Neurotransmitter Inhibition Activity

#### 2.7.1. AChE (Acetylcholinesterase Enzyme) Inhibition of a Functional Cone Mushroom (FCM) Snack Bar

The activity of AChE suppression was evaluated utilizing the colorimetric technique [5]. In summary, 25 µL of the sample was incubated for 5 min with a reaction mixture comprising 25 µL of 15 mM acetyl thiocholine iodide (ATCI), 75 µL of 5,5′-dithio-bis-2-nitrobenzoic acid (DTNB) and 50 µL of Tris-HCl (pH 8.0). The absorbance at 412 nm was measured using a microplate reader before and after the addition of 0.22 U/mL of 25 µL of AChE derived from whole brain homogenate. The inhibition percentage was determined by comparing the hydrolysis rate of ATCI in the samples with that of the blank (Tris-buffer). Donepezil (ARICEPT^®^, New York, NY, USA) served as the reference standard. The activity of AChE inhibition is quantified by the IC50 value. Each sample was conducted in triplicate.

#### 2.7.2. MAO (Monoamine Oxidase Enzyme) Inhibition of a Functional Cone Mushroom (FCM) Snack Bar

The activity of MAO inhibition was assessed utilizing the colorimetric technique. The chromogenic solution formulated for the assay mixture of 1 mM vanillic acid, 500 µM 4-aminoantipyrine and peroxidase (Sigma-Aldrich, Saint Louis, MO, USA) in a potassium phosphate buffer (0.2 M, pH 7.6). A homogenate was produced in 0.1 M potassium phosphate buffer, pH 7.4 (1:5 *w*/*v*), then centrifuged at 12,000 rpm for 10 min at 4 °C. The supernatant was obtained and utilized as the source of monoamine oxidase. The assay combination comprised 25 µL of monoamine oxidase, 25 µL of extracts at varying doses, 50 µL of chromogenic solution and 200 µL of 500 µM P-Tyramine. The reaction mixture was incubated for 30 min at 37 °C following mixing, and the absorbance was assessed at 490 nm [5]. The results are expressed as IC50 values.

### 2.8. Study Design and Participants

A randomized, double-blind, placebo-controlled study was performed for 6 weeks to determine the effect of a functional cone mushroom (FCM) snack bar on cognitive processing. This study was approved by the Institutional Review Board of Khon Kaen University Ethics Committee for Human Research, Khon Kaen Province, Thailand (HE661264), and was consistent with the Declaration of Helsinki. The protocol was also registered with the Thai Clinical Trials Registry (TCTR20230613004). A total of 26 healthy 45–60-year-old males and females were recruited and signed consent forms before participating in this study. Participants were randomly assigned to placebo, FCM1 (consuming a snack bar containing functional protein concentrate derived from cone mushroom at a dose of 1 g/serving/day), or FCM2 (consuming a snack bar containing functional protein concentrate derived from cone mushroom at a dose of 2 g/serving/day). The placebo contained all the same ingredients as the preparation of the snack bar containing protein concentrate powder derived from cone mushroom but without a functional cone-mushroom-derived protein concentrate. The chosen dose for this study was based on an in vitro study of biological activity related to acetylcholinesterase inhibition. The placebo and snack bar for consumption had the same appearance and taste. Demographic data, body composition and cognitive function and memory were evaluated at baseline and at the end of the study period (6 weeks). Dietary intake, physical activity and adverse events were recorded with a weekly diet and physical activity form, 24 h dietary recall form and adverse event form to assess the daily diet and physical activity alongside any side effects of the intervention.

### 2.9. Sample Size Calculation

The calculation of the sample size was performed using 80% power and a 95% level of confidence by the comparing two means method [33]. This calculation was based on similar research carried out in a study of cognitive function [34], with N being 8–9/group.

### 2.10. Cognitive Function Assessment

A study evaluated cognitive function with a non-invasive event-related potential (ERP) methodology. The procedures are elaborated in an earlier paper [5]. Brain activity was assessed via Ag–AgCl disk electrodes placed on the scalp, following the worldwide 10/20 technique. Participants were directed to distinguish between two audio stimuli, 650 Hz and 1 kHz, categorizing them as targeted and non-targeted stimuli. The investigation utilized Scan 4.3 software, focusing on variations in latency and peak amplitude of N100 and P300 at the Fz and Cz sites. N100 was a negative peak occurring between 65 and 135 milliseconds, whereas P300 was a positive peak occurring between 280 and 350 milliseconds.

### 2.11. Computerized Assessment Battery Test

The computerized assessment battery test was performed using a computer with a high-resolution VGA color monitor and, with the exception of written word recall tests. The test responses were recorded via two-button (yes/no) response box. Tests were monitored in the following order as previously mentioned by our research team [35]:

Word presentation: The participant has to remember fifteen words, matched for frequency and concreteness as sequencely presented on the monitor. The stimulus duration was 1 s, as was the interstimulus interval.

Picture presentation: The participants were presented with a series of 20 photographic images on a monitor at a rate of 1 every 3 s, with a stimulus duration of 1 s.

Simple reaction time: The participants were instructed to press the ‘yes’ response button as quickly as possible every time the word ‘yes’ was presented on the monitor. The task measures accuracy (%), reaction time (milliseconds) and number of false alarms.

Digit vigilance task: A target digit was randomly selected and constantly displayed to the right of the monitor screen. A series of digits were presented in the center of the screen at the rate of 80 min−1 and the participant was required to press the ‘yes’ button as quickly as possible every time the digit in the series matched the target digit. The task lasted 1 min and there were 15 stimulus–target matches. Task measures were accuracy (%), reaction time (milliseconds) and number of false alarms.

Choice reaction time: The choice reaction time involves either the word ‘no’ or ‘yes’ being presented on the monitor and the participant being required to press the corresponding button as quickly as possible. Reaction times (milliseconds) and accuracy (%) were recorded.

Spatial working memory: The spatial working memory involved participants memorizing the position of illuminated windows in a house, and then being asked to press the ‘yes’ or ‘no’ response button as quickly as possible. Mean reaction times were measured in milliseconds and the accuracy of responses to both original and novel (distractor) stimuli were recorded as percentages that were used to derive a percentage greater than chance performance score.

Numeric working memory: The numerical working memory involved participants holding five digits sequentially and deciding whether they had been in the original series. The mean reaction times were measured in milliseconds, and the accuracy of responses to both original and novel stimuli was recorded as percentages.

### 2.12. Statistical Analysis

All data are presented as the mean ± standard error of the mean (SEM). All data were analyzed by comparison between the placebo and experimental groups (FCM1 and FCM2). The data were also compared between baseline and after a 6-week consumption period. The Kolmogorov–Smirnov test was used to analyze the normality of the data. Statistical analysis was conducted using ANOVA, followed by the post hoc Tukey test. Substantial differences among groups were considered when the *p*-value < 0.05.

## 3. Results

### 3.1. Amino Acid Profile of Cone Mushroom and Cone Mushroom Protein Concentrate

The total volumes of essential amino acids (EAAs) and non-essential amino acids (NEAAs) were compared for the cone mushroom and the cone mushroom protein concentrate. The cone mushroom contains a higher total volume of EAAs and NEAAs than the cone mushroom protein concentrate, as shown in Table 1.

### 3.2. Amino Acid Profile of FCM Snack Bar

We developed a snack bar containing cone mushrooms, almonds, watermelon seeds, pumpkin seeds, cashew nuts and other fruits. The evaluation results regarding essential amino acids in the FCM snack bar showed that the three highest contents of essential amino acids were those for arginine, leucine and valine, with quantities of 1345.53, 823.07 and 646.21 g/100 g sample, respectively, as shown in Table 2.

### 3.3. Nutritional Content of the FCM Snack Bar

The FCM snack bar contained 448.09 kcal of energy per 100 g, with 13.97 g of protein, 48.17 g of carbohydrates, 22.17 g of fat and 16.63 g of dietary fiber, as shown in Table 3.

### 3.4. Phytochemical Contents of the FCM Snack Bar

The total phenolic compound contents in the placebo and FCM were 1.94 ± 0.14 and 2.29 ± 0.15 mg of gallic acid/g of sample, respectively. The total flavonoid contents in the placebo and FCM snack bar were 0.08 ± 0.01 and 0.13 ± 0.01 mg of quercetin/g, respectively. The total flavonoid content was significantly higher in the FCM snack bar than in the placebo (*p* = 0.0257), as shown in Table 4.

### 3.5. Biological Activities of the FCM Snack Bar

To evaluate the antioxidant activity of the FCM snack bar, DPPH and ABTS inhibition tests were performed. The percentages of inhibition of DPPH and ABTS were 16.84 ± 0.26 and 19.40 ± 0.06, respectively. In addition, COX-II inhibition was tested to examine anti-inflammatory activity. The percentage of inhibition of COX-II was 12.78 ± 0.10. Acetylcholinesterase inhibition and monoamine oxidase inhibition were evaluated to demonstrate the neuroprotective activity of the FCM snack bar. The percentages of acetylcholinesterase and monoamine oxidase suppression were 20.37 ± 0.02 and 18.97 ± 0.05, respectively (Table 5).

### 3.6. Effect of the FCM Snack Bar Depending on Subjects’ Demographic Data

The demographic data, including age, gender, body temperature, heart rate, systolic and diastolic blood pressure, body weight and body mass index (BMI), at baseline and 6 weeks of intervention are presented in Table 6 and Table 7, respectively. There were no significant differences in physiological parameters throughout the study period (*p* > 0.05).

### 3.7. Effect of the FCM Snack Bar on Body Composition

The body compositions of the participants at baseline and after a 6-week consumption period are shown in Table 8. No significant changes in body composition parameters were observed in any of the studied groups (*p* > 0.05).

### 3.8. Effect of the FCM Snack Bar on Cognitive Processing

To evaluate the effect of the functional cone mushroom protein snack bar on cognitive processing, a non-invasive event-related potential (ERP) assessment was performed. At baseline, there were no significant differences in the latency or amplitude of N100 and P300 in all groups. After 6 weeks of intervention, a significant increase in N100 amplitude at the Fz location was observed in participants who consumed the functional cone mushroom protein snack bar at a dose of 1 g compared to the placebo group (*p* = 0.015). Additionally, subjects who consumed the functional cone mushroom protein snack bar at a dose of 2 g showed a significantly increased P300 amplitude compared to those in the placebo group (*p* = 0.044) (Table 9).

### 3.9. Effect of the FCM Snack Bar on Memory

To evaluate the effect of the functional cone mushroom protein snack bar on memory, a computerized assessment battery test (CDR) assessment was evaluated. At baseline, there were no significant differences in any cognitive domains of memory tests. Interestingly, an increased percent accuracy of numeric working memory was observed in subjects who consumed the functional cone mushroom protein snack bar at a dose of 2 g compared to those in the placebo group (*p* = 0.048) (Table 10).

## 4. Discussion

This study revealed the richness of essential amino acids, dietary fiber and flavonoids in a functional cone mushroom protein snack bar. The present study also demonstrated that daily consumption of the functional cone mushroom protein snack bar for 6 weeks increased the N100 and P300 amplitude at the Fz location and enhanced %accuracy of numeric working memory, indicating improvements in cognitive function and memory. The proposed mechanism may involve antioxidant activity along with AChE and MAO suppression activity.

The aim of the first part of this study was to formulate and evaluate the amino acid profiles, phytochemical contents and biological activities of a functional protein snack bar enriched with cone mushroom. As lower essential amino acid and non-essential amino acid contents are observed in cone mushroom protein compared to other protein concentrates, we decided to develop a functional cone mushroom protein snack bar by mixing cone mushroom with almonds, watermelon seeds, pumpkin seeds, cashew nuts and other fruits. The developed functional cone mushroom protein snack bar showed higher levels of total protein, dietary fiber and essential and non-essential amino acids than a cone mushroom protein concentrate.

A phytochemical content analysis of the functional cone mushroom protein snack bar revealed high total flavonoid contents, which may indicate the antioxidant activity of this product [36]. To evaluate the antioxidant activity, DPPH and ABTS inhibition tests were performed. We found that the functional protein snack bar enriched with cone mushroom suppressed both DPPH and ABTS, confirming its antioxidant activity. It is well-accepted that flavonoids exert antioxidant activity by their direct scavenging activity and transferring hydrogen atoms to free radicals [19]. Furthermore, we tested the snack bar’s neuroprotective activity by assessing the inhibition of AChE and MAO. This developed product also showed a high capacity for AChE suppression, with a lesser effect on MAO.

The demographic data and body composition of the participants did not change following the consumption of the functional cone mushroom protein snack bar for 6 weeks. All parameters were within the normal physiological range at the end of the study. The placebo and snack bar products showed similar caloric energy levels. Additionally, there was no significant difference in dietary intake or physical activity in the placebo and treatment volunteers throughout the study period. This finding indicates that this product did not cause metabolic alterations.

Regarding the beneficial health effects of the functional cone mushroom protein snack bar, we conducted a clinical study to evaluate the impact of the developed product on cognitive function and related mechanisms. The cognitive function was assessed via the ERP at baseline and at the end of treatment. At baseline, we did not observe any significant differences in N100 or P300 latency or amplitude in either of the studied groups, indicating the same cognitive function as at the beginning time point. Interestingly, a significant increase in N100 and P300 amplitude and accuracy of numeric working memory following continuous consumption of the functional cone mushroom protein snack bar was observed, which may indicate an enhancement in cognitive processing and working memory among middle-aged individuals. The N100 wave is evoked mainly by sensory neurons related to attention and sensory processing, which have been localized to the auditory cortex, with additional contributions from the frontal cortex and parietal sources [37,38]. It is widely accepted that the N100 amplitude represents an enhancement in the synchronization of functional neurons during selective attention. Additionally, N100 has been associated with improved memory, including short-term memory. The P300 amplitude is usually evoked by the Oddball paradigm, with the highest amplitude usually detected from the central part of the scalp and the parietal lobe area [39]. The P300 amplitude is associated with cognitive processing, including attention, evaluative processing, judgment, working memory capacity and decision making [40,41,42,43]. A reduction in P300 and N100 amplitudes is associated with several neurodegenerative diseases, such as dementia, Alzheimer’s disease, Parkinson’s disease and depression [34,44,45,46]. Several studies reported that reduced amplitudes of P300 and N100 may be a useful marker for the early detection of cognitive impairment. An increase in N100 and P300 amplitudes along with the accuracy of numeric working memory may reflect an improvement in the information encoding process, resulting in focused and enhanced attention and, consequently, enhanced cognitive performance and memory in middle-aged adults. Therefore, the alteration of ERP amplitudes could indicate the cognition-enhancing effects of the functional ingredients in the *Termitomyces fuliginosus* protein bar and may be considered a recovery marker following therapeutic interventions. Similar findings have been reported, showing the impact of various types of mushroom supplementation on cognitive function improvement in both healthy volunteers and patients with various age-related neurodegenerative disorders such as Alzheimer’s disease, depression and anxiety [14,47,48]. However, most studies involved longer treatment periods than this study [47]. We suggest that the cognitive improvement effect of *Termitomyces fuliginosus* begins at week 6 after supplementation. It is well known that several factors affect cognitive function in humans, including age, gender and health factors such as physical activity, drinking and smoking, history of disease and BMI [49,50]. According to the inclusion and exclusion criteria of this study, all of the mentioned confounding factors were similar across all the treated groups, thus, it is unlikely that any expectancy impacts influenced the improvement in cognitive function.

Regarding the underlying mechanism, ACh is considered a neurotransmitter involving cognitive and memory processing [19]. This neurotransmitter is normally broken down by AChE enzymes. The functional *Termitomyces fuliginosus* protein snack bar exhibited an AChE inhibition effect, which may increase both the level and duration of action of acetylcholine at the neuromuscular junctions. MAO is an enzyme responsible for the oxidative deamination of monoamine neurotransmitters like serotonin, dopamine and norepinephrine; the activation of MAO results in decreased availability of these neurotransmitters, which are essential for neuronal transmission and thereby influencing mood, and cognition [51]. Reduced levels of these neurotransmitters are associated with the pathophysiology of various neurodegenerative diseases, while MAO inhibitors have been discovered for the treatment of some neurodegenerative diseases [52,53]. According to the biological activity tests, the developed product showed a high capacity for AChE suppression with a lesser effect on MAO inhibition. We hypothesize that the potential synergistic effects of inhibiting both AChE and MAO may contribute to the underlying mechanism of the cognition-enhancing effects of *Termitomyces fuliginosus*. Similar results regarding AChE and MAO suppression activity of the various types of mushrooms have been observed in previous studies [19,54,55,56]. As such, future investigation of neurotransmitter levels, especially Ach and monoamine neurotransmitters, in biological samples should be performed to confirm the cognitive-modifying effects of *Termitomyces fuliginosus*. Furthermore, it is well known that *Termitomyces fuliginosus* contains dietary fiber, which has a beneficial effect on the gut–brain axis [57]. The developed *Termitomyces fuliginosus* protein bar contains a high level of dietary fiber, which may increase activity in the gut microbiome. However, due to the richness of the total flavonoid content and high antioxidant activity, the antioxidant effect of this developed product cannot be excluded. Previous studies have reported that supplementation with flavonoid-rich products is associated with enhanced neuroplasticity and improved brain health. Flavonoid metabolites can cross the blood–brain barrier and can localize in brain regions related to cognitive function [28,58]. Moreover, flavonoids are known to improve blood flow to the brain, facilitating better oxygen and nutrient delivery [29]. Additionally, dietary flavonoids exert probiotic activity and can alter the gut microbiome, supporting a beneficial effect on the gut–brain axis and maintaining cognitive function as well [25,58]. Our investigation aligns with previous studies that demonstrate the neuroprotective effects of flavonoids, particularly in improving cognitive performance and reducing oxidative stress. Several studies have reported that flavonoid-rich supplementation, such as berries and oranges, is associated with enhanced memory and cognitive function [59,60,61]. Further analysis of the flavonoid metabolites and their anti-inflammation and antioxidant activity in participants should be investigated. This aspect still requires further research to confirm the mechanism of action. Additionally, a larger study population and a longer period of assessment are needed to further evaluate the effects of *Termitomyces fuliginosus* on cognitive function and memory in middle-aged adults.

## 5. Conclusions

This research data clearly demonstrates that consuming a functional cone mushroom (FCM) protein snack bar daily for 6 weeks can improve cognitive function and working memory. The possible underlying mechanisms may involve flavonoid-related antioxidant and AChE and MAO suppression activity.

## Figures and Tables

**Table 1:** Amino acid profiles of the cone mushroom and cone-mushroom-derived protein concentrate.

Amino Acid Profile	Cone Mushroom	Cone Mushroom Protein Concentrate
	Mg/100 g Protein	
**EAAs**		
Arginine	4542.52	ND
Histidine	2098.65	1827.61
Isoleucine	2885.66	3414.04
Leucine	5842.37	2810.14
Lysine	5399.42	3252.26
Methionine	829.49	ND
Phenylalanine	3445.22	<250.00
Threonine	4037.81	ND
Tryptophan	1898.25	<150.00
Valine	4717.77	5559.13
**Total EAA**	**35,697.15**	**16,863.18**
**NEAAs**		
Alanine	5853.87	6255.23
Aspartic acid	7941.97	6591.89
Cysteine	-	-
Cystine	<200.00	ND
Glutamic acid	14,134.23	12,284.87
Glycine	3897.99	5124.49
Hydroxyproline	ND	ND
Proline	3435.40	3519.97
Serine	4427.12	<200.00
Tyrosine	2374.09	ND
**Total NEAAs**	**42,064.67**	**33,776.44**

EAAs: essential amino acids, NEAAs: non-essential amino acids, ND: not detected, -: not measured.

**Table 2:** Amino acid profile of FCM snack bar.

Amino Acid Profile	FCM Snack Bar (mg/100 g Sample)
**EAAs**	
Arginine	1345.53
Histidine	307.23
Isoleucine	431.27
Leucine	823.07
Lysine	468.21
Methionine	ND
Phenylalanine	573.22
Threonine	425.86
Tryptophan	153.72
Valine	646.21
**Total EAA**	**5174.32**

EAAs: essential amino acids, ND: not detected.

**Table 3:** Nutritional information on the FCM snack bar.

Parameters	100 g of Placebo	100 g of FCM	Method
Energy (kcal)	452.57	448.09	Compendium of Method for Food Analysis (2003), p. 2–18
Protein (g)	14.11	13.97	AOAC (2019) 981.10
Carbohydrate (g)	48.65	48.17	Compendium of Method for Food Analysis (2003), p. 2–9 to p. 2–10
Total fat (g)	22.39	22.17	AOAC (2019) 922.06
Dietary fiber (g)	16.80	16.63	In-house method TE-CH-076 based on AOAC (2019) 985.29

**Table 4:** Phytochemical contents in the FCM snack bar.

Phytochemical Contents	Placebo	FCM Snack Bar
Total phenolic content (mg of gallic acid/g)	1.94 ± 0.14	2.29 ± 0.15
Total flavonoid content (mg of quercetin/g)	0.08 ± 0.01	0.13 ± 0.01 *

Data are presented as mean ± SEM. * *p* < 0.05 compared to the placebo.

**Table 5:** Biological activities of the FCM snack bar.

Biological Activities Parameters	Placebo		FCM Snack Bar	
	% Inhibition	IC50 (mg/mL)	% Inhibition	IC50 (mg/mL)
DPPH inhibition	14.83 ± 0.27	8.64 ± 0.04	16.84 ± 0.26 **	7.87 ± 0.04
ABTS inhibition	17.30 ± 0.11	5.58 ± 0.02	19.40 ± 0.06 ***	4.38 ± 0.01
COX-II inhibition	10.35 ± 0.10	6.89 ± 0.03	12.78 ± 0.10 ***	5.38 ± 0.06
Acetylcholinesterase inhibition	17.95 ± 0.06	5.76± 0.02	20.37 ± 0.02 ***	4.48 ± 0.02
Monoamine oxidase inhibition	18.26 ± 0.05	5.79 ± 0.01	18.97 ± 0.05 *	5.23 ± 0.03

DPPH: 1,1-diphenyl-2-picrylhydrazyl radical, ABTS: 2,2′-azino-bis-(3-ethylbenzthiazoline-6-sulphonic acid radical, COX-II: Cyclooxygenase-II. Data are presented as mean ± SEM. * *p*-values < 0.05, ** *p*-values < 0.01, *** *p* < 0.001 compared to the placebo.

**Table 6:** Physiological parameters of participants at baseline.

Parameters	Baseline		
	Placebo (*n* = 8)	FCM1 (*n* = 9)	FCM2 (*n* = 9)
Age (year)	52.50 ± 1.91	50.11 ± 1.49	52.33 ± 1.03
Gender (male/female)	0/8	2/7	1/8
Blood Temperature (°C)	36.58 ± 0.05	36.58 ± 0.05	36.54 ± 0.05
Heart rate (beats/min)	69.63 ± 2.97	66.78 ± 1.94	70.56 ± 1.94
Respiratory rate (breaths/min)	17.00 ± 0.27	17.00 ± 0.17	17.1 ± 0.20
Systolic BP (mmHg)	115.75 ± 2.70	115.89 ± 3.60	115.22 ± 3.15
Diastolic BP (mmHg)	76.75 ± 2.72	76.22 ± 3.13	73.22 ± 2.40
Body weight (kg)	57.33 ± 4.52	59.71 ± 2.88	62.14 ± 5.80
BMI (kg/m^2^)	24.18 ± 1.88	23.12 ± 0.89	24.74 ± 1.60

Data are presented as mean ± SEM.

**Table 7:** Physiological parameters of participants after a 6-week consumption period.

Parameters	6 Weeks		
	Placebo (*n* = 8)	FCM1 (*n* = 9)	FCM2 (*n* = 9)
Age (year)	52.50 ± 1.91	50.11 ± 1.49	52.33 ± 1.03
Gender (male/female)	0/8	2/7	1/8
Blood temperature (°C)	36.60 ± 0.05	36.57 ± 0.06	36.56 ± 0.05
Heart rate (beats/min)	73.75 ± 2.07	70.22 ± 1.73	74.00 ± 2.46
Respiratory rate (breaths/min)	17.13 ± 0.23	17.11 ± 0.11	17.67 ± 0.17
Systolic BP (mmHg)	110.63 ± 4.93	116.89 ± 4.15	110.00 ± 3.08
Diastolic BP (mmHg)	71.38 ± 3.36	72.44 ± 2.92	69.67 ± 2.86
Body weight (kg)	56.94 ± 4.50	59.49 ± 2.88	62.20 ± 5.71
BMI (kg/m^2^)	24.02 ± 1.85	23.02 ± 0.87	24.76 ± 1.53

Data are presented as mean ± SEM.

**Table 8:** Body composition of participants at baseline and after a 6-week consumption period.

Parameters		Placebo (*n* = 8)	FCM1 (*n* = 9)	FCM2 (*n* = 9)
Water (%)	Baseline	48.25 ± 1.86	51.42 ± 2.02	49.52 ± 1.70
	6 weeks	48.39 ± 1.87	51.58 ± 2.02	49.17 ± 1.56
Visceral Fat (%)	Baseline	6.63 ± 1.08	7.00 ± 0.55	7.78 ± 1.51
	6 weeks	6.50 ± 1.05	6.89 ± 0.61	7.78 ± 1.51
Total Fat (%)	Baseline	34.11 ± 2.55	29.74 ± 2.76	32.37 ± 2.32
	6 weeks	33.89 ± 2.55	29.53 ± 2.75	32.84 ± 2.13
Muscle Mass (%)	Baseline	34.93 ± 1.30	39.38 ± 1.95	39.18 ± 3.38
	6 weeks	34.79 ± 1.31	39.32 ± 1.92	39.07 ± 3.42
Muscle Mass Fat (%)	Baseline	4.25 ± 0.31	4.33 ± 0.37	4.33 ± 0.33
	6 weeks	4.13 ± 0.40	4.44 ± 0.38	4.33 ± 0.33
Bone Mass (%)	Baseline	2.09 ± 0.12	2.34 ± 0.12	2.36 ± 0.19
	6 weeks	2.06 ± 0.11	2.34 ± 0.12	2.33 ± 0.19
BMR (kcal)	Baseline	1098.00 ± 53.44	1206.56 ± 52.42	1219.78 ± 101.28
	6 weeks	1093.13 ± 53.53	1204.00 ± 51.49	1215.11 ± 102.26

Data are presented as mean ± SEM.

**Table 9:** Effect of the functional cone mushroom protein snack bar on the latency and amplitude of N100 and P300 waves of event-related potential at baseline and after a 6-week consumption period.

	Parameters		Placebo (*n* = 8)	FCM1 (*n* = 9)	FCM2 (*n* = 9)
Fz	N100 Latency (ms)	Baseline	108.38 ± 1.57	106.11 ± 2.00	105.78 ± 2.27
		6 weeks	110.63 ± 1.98	110.44 ± 1.69	109.44 ± 1.90
	N100 Amplitude (μV)	Baseline	5.25 ± 0.79	6.68 ± 0.88	4.98 ± 0.49
		6 weeks	5.36 ± 0.76	7.92 ± 0.61 *	6.56 ± 0.63
	P300 Latency (ms)	Baseline	342.63 ± 7.02	337.00 ± 4.72	348.33 ± 3.94
		6 weeks	336.13 ± 3.33	343.56 ± 3.21	343.44 ± 2.33
	P300 Amplitude (μV)	Baseline	25.41 ± 2.36	25.13 ± 2.73	29.34 ± 2.41
		6 weeks	25.35 ± 2.85	24.52 ± 1.24	31.63 ± 1.86 *
Cz	N100 Latency (ms)	Baseline	111.75 ± 1.75	106.56 ± 1.82	106.25 ± 2.05
		6 weeks	107.13 ± 1.47	107.78 ± 1.34	106.78 ± 1.75
	N100 Amplitude (μV)	Baseline	6.44 ± 0.88	7.11 ± 0.94	4.89 ± 0.95
		6 weeks	4.94 ± 0.81	5.37 ± 0.80	5.64 ± 0.70
	P300 Latency (ms)	Baseline	344.75 ± 4.05	334.22 ± 2.85	343.56 ± 3.00
		6 weeks	342.63 ± 5.97	345.44 ± 4.73	343.44 ± 2.71
	P300 Amplitude (μV)	Baseline	26.35 ± 3.23	22.92 ± 2.58	26.36 ± 2.43
		6 weeks	25.25 ± 2.81	24.64 ± 2.26	24.84 ± 1.48

Data are presented as mean ± SEM, * *p*-value < 0.05 compared to the placebo.

**Table 10:** Effect of the functional cone mushroom protein snack bar on the memory test by computerized assessment battery test at baseline and after a 6-week consumption period.

Cognitive Domains	Parameters		Placebo (*n* = 8)	FCM1 (*n* = 9)	FCM2 (*n* = 9)
**Word** **Recognition**	Time	Baseline	1255.88 ± 97.31	1290.08 ± 105.64	1169.48 ± 34.27
		6 weeks	1118.16 ± 47.20	1152.69 ± 57.01	1195.09 ± 76.90
	%Accuracy	Baseline	90.42 ± 2.13	82.96 ± 4.17	90.00 ± 2.94
		6 weeks	94.17 ± 1.64	88.89 ± 4.12	92.59 ± 1.82
**Picture** **Recognition**	Time	Baseline	1436.75 ± 109.25	1379.25 ± 72.31	1264.16 ± 43.07
		6 weeks	1184.33 ± 64.37	1274.51 ± 59.56	1224.64 ± 78.72
	%Accuracy	Baseline	85.00 ± 2.31	88.13 ± 2.49	89.34 ± 1.99
		6 weeks	85.00 ± 1.71	86.11 ± 2.98	87.78 ± 2.37
**Sample** **reaction**	Time	Baseline	691.05 ± 46.18	779.40 ± 59.04	713.88 ± 25.92
		6 weeks	659.08 ± 39.96	767.54 ± 50.66	669.92 ± 29.11
**Digit** **vigilance**	Time	Baseline	688.10 ± 13.04	679.07 ± 15.76	702.84 ± 9.89
		6 weeks	687.269 ± 17.38	718.71 ± 24.03	703.85 ± 9.98
	%Accuracy	Baseline	90.55 ± 1.86	91.03 ± 2.07	91.45 ± 1.99
		6 weeks	93.43 ± 1.99	91.17 ± 2.20	92.88 ± 1.37
**Choice** **reaction time**	Time	Baseline	935.92 ± 85.49	968.16 ± 84.15	904.65 ± 32.62
		6 weeks	879.90 ± 62.53	912.64 ± 59.01	843.24 ± 24.81
	%Accuracy	Baseline	90.55 ± 1.86	91.03 ± 2.07	91.45 ± 1.99
		6 weeks	93.43 ± 1.99	91.17 ± 2.20	92.88 ± 1.37
**Spatial memory**	Time	Baseline	1467.67 ± 110.03	1550.50 ± 119.26	1325.98 ± 86.67
		6 weeks	1249.20 ± 102.01	1326.77 ± 68.70	1316.46 ± 76.29
	%Accuracy	Baseline	89.93 ± 3.82	90.74 ± 3.07	91.73 ± 3.82
		6 weeks	95.49 ± 2.16	93.21 ± 3.68	92.90 ± 3.21
**Numeric working memory**	Time	Baseline	1060.52 ± 45.86	1148.97 ± 49.17	1051.99 ± 39.83
		6 weeks	1048.26 ± 61.02	1176.04 ± 51.13	1074.11 ± 41.51
	%Accuracy	Baseline	90.42 ± 4.86	87.41 ± 5.35	95.56 ± 2.08
		6 weeks	91.43 ± 2.39	93.33 ± 3.39	98.75 ± 0.60 *

Data are presented as mean ± SEM, * *p*-value < 0.05 compared to the placebo.

## Data Availability

Data are contained within the article and will be provided on request because all data are in the process of petty patent registration during this period.

## References

[1] Bai W., Chen P., Cai H., Zhang Q., Su Z., Cheung T., Jackson T., Sha S., Xiang Y.T. (2022). Worldwide prevalence of mild cognitive impairment among community dwellers aged 50 years and older: A meta-analysis and systematic review of epidemiology studies. Age Ageing.

[2] Pais R., Ruano L., Carvalho O.P., Barros H. (2020). Global Cognitive Impairment Prevalence and Incidence in Community Dwelling Older Adults—A Systematic Review. Geriatrics.

[3] Shiojima Y., Takahashi M., Takahashi R., Moriyama H., Bagchi D., Bagchi M., Akanuma M. (2022). Effect of Dietary Pyrroloquinoline Quinone Disodium Salt on Cognitive Function in Healthy Volunteers: A Randomized, Double-Blind, Placebo-Controlled, Parallel-Group Study. J. Am. Nutr. Assoc..

[4] Avgerinos K.I., Spyrou N., Bougioukas K.I., Kapogiannis D. (2018). Effects of creatine supplementation on cognitive function of healthy individuals: A systematic review of randomized controlled trials. Exp. Gerontol..

[5] Wattanathorn J., Somboonporn W., Thukham-Mee W., Sungkamnee S. (2022). Memory-Enhancing Effect of 8-Week Consumption of the Quercetin-Enriched Culinary Herbs-Derived Functional Ingredients: A Randomized, Double-Blind, Placebo-Controlled Clinical Trial. Foods.

[6] Vyas C.M., Manson J.E., Sesso H.D., Rist P.M., Weinberg A., Kim E., Moorthy M.V., Cook N.R., Okereke O.I. (2024). Effect of cocoa extract supplementation on cognitive function: Results from the clinic subcohort of the COSMOS trial. Am. J. Clin. Nutr..

[7] Dimopoulou M., Vareltzis P., Floros S., Androutsos O., Bargiota A., Gortzi O. (2023). Development of a Functional Acceptable Diabetic and Plant-Based Snack Bar Using Mushroom (*Coprinus comatus*) Powder. Foods.

[8] Singh A., Kumari A., Chauhan A.K. (2022). Formulation and evaluation of novel functional snack bar with amaranth, rolled oat, and unripened banana peel powder. J. Food Sci. Technol..

[9] Pinto V.R.A., Freitas T.B.O., Dantas M.I.S., Della Lucia S.M., Melo L.F., Minim V.P.R., Bressan J. (2017). Influence of package and health-related claims on perception and sensory acceptability of snack bars. Food Res. Int..

[10] Paloi S., Kumla J., Paloi B.P., Srinuanpan S., Hoijang S., Karunarathna S.C., Acharya K., Suwannarach N., Lumyong S. (2023). Termite Mushrooms (*Termitomyces*), a Potential Source of Nutrients and Bioactive Compounds Exhibiting Human Health Benefits: A Review. J. Fungi.

[11] Hsieh H.M., Ju Y.M. (2018). Medicinal components in Termitomyces mushrooms. Appl. Microbiol. Biotechnol..

[12] Park J.S., Kim Y.S., Kwon E., Yun J.W., Kang B.C. (2021). Genotoxicity Evaluation of Termite Mushroom, *Termitomyces albuminosus* (Agaricomycetes), Powder. Int. J. Med. Mushrooms.

[13] Sitati C.N.W., Ogila K.O., Waihenya R.W., Ochola L.A. (2021). Phytochemical Profile and Antimicrobial Activities of Edible Mushroom *Termitomyces striatus*. Evid. Based Complement. Alternat. Med..

[14] Docherty S., Doughty F.L., Smith E.F. (2023). The Acute and Chronic Effects of Lion’s Mane Mushroom Supplementation on Cognitive Function, Stress and Mood in Young Adults: A Double-Blind, Parallel Groups, Pilot Study. Nutrients.

[15] Cavanna F., Muller S., de la Fuente L.A., Zamberlan F., Palmucci M., Janeckova L., Kuchar M., Pallavicini C., Tagliazucchi E. (2022). Microdosing with psilocybin mushrooms: A double-blind placebo-controlled study. Transl. Psychiatry.

[16] Li I.C., Lee L.Y., Tzeng T.T., Chen W.P., Chen Y.P., Shiao Y.J., Chen C.C. (2018). Neurohealth Properties of *Hericium erinaceus* Mycelia Enriched with Erinacines. Behav. Neurol..

[17] Nagano M., Shimizu K., Kondo R., Hayashi C., Sato D., Kitagawa K., Ohnuki K. (2010). Reduction of depression and anxiety by 4 weeks Hericium erinaceus intake. Biomed. Res..

[18] Yang Y., Zhu D., Qi R., Chen Y., Sheng B., Zhang X. (2024). Association between Intake of Edible Mushrooms and Algae and the Risk of Cognitive Impairment in Chinese Older Adults. Nutrients.

[19] Nguyen T.K., Im K.H., Choi J., Shin P.G., Lee T.S. (2016). Evaluation of Antioxidant, Anti-cholinesterase, and Anti-inflammatory Effects of Culinary Mushroom *Pleurotus pulmonarius*. Mycobiology.

[20] Parikh V., Bangasser D.A. (2020). Cholinergic Signaling Dynamics and Cognitive Control of Attention. Curr. Top. Behav. Neurosci..

[21] Bernaud V.E., Hiroi R., Poisson M.L., Castaneda A.J., Kirshner Z.Z., Gibbs R.B., Bimonte-Nelson H.A. (2021). Age Impacts the Burden That Reference Memory Imparts on an Increasing Working Memory Load and Modifies Relationships With Cholinergic Activity. Front. Behav. Neurosci..

[22] Ashby F.G., Zetzer H.A., Conoley C.W., Pickering A.D. (2024). Just do it: A neuropsychological theory of agency, cognition, mood, and dopamine. J. Exp. Psychol. Gen..

[23] Rai S.N., Mishra D., Singh P., Vamanu E., Singh M.P. (2021). Therapeutic applications of mushrooms and their biomolecules along with a glimpse of in silico approach in neurodegenerative diseases. Biomed. Pharmacother..

[24] Szucko-Kociuba I., Trzeciak-Ryczek A., Kupnicka P., Chlubek D. (2023). Neurotrophic and Neuroprotective Effects of *Hericium erinaceus*. Int. J. Mol. Sci..

[25] Magni G., Riboldi B., Petroni K., Ceruti S. (2022). Flavonoids bridging the gut and the brain: Intestinal metabolic fate, and direct or indirect effects of natural supporters against neuroinflammation and neurodegeneration. Biochem. Pharmacol..

[26] Polyiam P., Thukhammee W. (2024). A Comparison of Phenolic, Flavonoid, and Amino Acid Compositions and In Vitro Antioxidant and Neuroprotective Activities in Thai Plant Protein Extracts. Molecules.

[27] Minocha T., Birla H., Obaid A.A., Rai V., Sushma P., Shivamallu C., Moustafa M., Al-Shehri M., Al-Emam A., Tikhonova M.A. (2022). Flavonoids as Promising Neuroprotectants and Their Therapeutic Potential against Alzheimer’s Disease. Oxid. Med. Cell Longev..

[28] Calis Z., Mogulkoc R., Baltaci A.K. (2020). The Roles of Flavonols/Flavonoids in Neurodegeneration and Neuroinflammation. Mini Rev. Med. Chem..

[29] Wood E., Hein S., Mesnage R., Fernandes F., Abhayaratne N., Xu Y., Zhang Z., Bell L., Williams C., Rodriguez-Mateos A. (2023). Wild blueberry (poly)phenols can improve vascular function and cognitive performance in healthy older individuals: A double-blind randomized controlled trial. Am. J. Clin. Nutr..

[30] Liuzzi G.M., Petraglia T., Latronico T., Crescenzi A., Rossano R. (2023). Antioxidant Compounds from Edible Mushrooms as Potential Candidates for Treating Age-Related Neurodegenerative Diseases. Nutrients.

[31] Quettier-Deleu C., Gressier B., Vasseur J., Dine T., Brunet C., Luyckx M., Cazin M., Cazin J.C., Bailleul F., Trotin F. (2000). Phenolic compounds and antioxidant activities of buckwheat (*Fagopyrum esculentum* Moench) hulls and flour. J. Ethnopharmacol..

[32] Wattanathorn J., Kawvised S., Thukham-Mee W. (2019). Encapsulated Mulberry Fruit Extract Alleviates Changes in an Animal Model of Menopause with Metabolic Syndrome. Oxid. Med. Cell Longev..

[33] Sakpal T.V. (2010). Sample size estimation in clinical trial. Perspect. Clin. Res..

[34] Hunerli D., Emek-Savas D.D., Cavusoglu B., Donmez Colakoglu B., Ada E., Yener G.G. (2019). Mild cognitive impairment in Parkinson’s disease is associated with decreased P300 amplitude and reduced putamen volume. Clin. Neurophysiol..

[35] Peth-Nui T., Wattanathorn J., Muchimapura S., Tong-Un T., Piyavhatkul N., Rangseekajee P., Ingkaninan K., Vittaya-Areekul S. (2012). Effects of 12-Week Bacopa monnieri Consumption on Attention, Cognitive Processing, Working Memory, and Functions of Both Cholinergic and Monoaminergic Systems in Healthy Elderly Volunteers. Evid. Based Complement. Alternat. Med..

[36] Molole G.J., Gure A., Abdissa N. (2022). Determination of total phenolic content and antioxidant activity of *Commiphora mollis* (Oliv.) Engl. resin. BMC Chem..

[37] Duncan E., Roach B.J., Massa N., Hamilton H.K., Bachman P.M., Belger A., Carrion R.E., Johannesen J.K., Light G.A., Niznikiewicz M.A. (2022). Auditory N100 amplitude deficits predict conversion to psychosis in the North American Prodrome Longitudinal Study (NAPLS-2) cohort. Schizophr. Res..

[38] Naatanen R., Picton T. (1987). The N1 wave of the human electric and magnetic response to sound: A review and an analysis of the component structure. Psychophysiology.

[39] Zhang Y., Yang T., He Y., Meng F., Zhang K., Jin X., Cui X., Luo X. (2023). Value of P300 amplitude in the diagnosis of untreated first-episode schizophrenia and psychosis risk syndrome in children and adolescents. BMC Psychiatry.

[40] Dallmer-Zerbe I., Popp F., Lam A.P., Philipsen A., Herrmann C.S. (2020). Transcranial Alternating Current Stimulation (tACS) as a Tool to Modulate P300 Amplitude in Attention Deficit Hyperactivity Disorder (ADHD): Preliminary Findings. Brain Topogr..

[41] Thomaschewski M., Heldmann M., Uter J.C., Varbelow D., Munte T.F., Keck T. (2021). Changes in attentional resources during the acquisition of laparoscopic surgical skills. BJS Open.

[42] Toyoshima K., Toyomaki A., Miyazaki A., Martinez-Aran A., Vieta E., Kusumi I. (2020). Associations between cognitive impairment and P300 mean amplitudes in individuals with bipolar disorder in remission. Psychiatry Res..

[43] Herzog N.D., Steinfath T.P., Tarrasch R. (2021). Critical Dynamics in Spontaneous Resting-State Oscillations Are Associated With the Attention-Related P300 ERP in a Go/Nogo Task. Front. Neurosci..

[44] Santopetro N.J., Brush C.J., Bruchnak A., Klawohn J., Hajcak G. (2021). A reduced P300 prospectively predicts increased depressive severity in adults with clinical depression. Psychophysiology.

[45] Dan Z., Li H., Xie J. (2023). Efficacy of donepezil plus hydrogen-oxygen mixture inhalation for treatment of patients with Alzheimer disease: A retrospective study. Medicine.

[46] Tarawneh H.Y., Mulders W., Sohrabi H.R., Martins R.N., Jayakody D.M.P. (2021). Investigating Auditory Electrophysiological Measures of Participants with Mild Cognitive Impairment and Alzheimer’s Disease: A Systematic Review and Meta-Analysis of Event-Related Potential Studies. J. Alzheimers Dis..

[47] Li I.C., Chang H.H., Lin C.H., Chen W.P., Lu T.H., Lee L.Y., Chen Y.W., Chen Y.P., Chen C.C., Lin D.P. (2020). Prevention of Early Alzheimer’s Disease by Erinacine A-Enriched *Hericium erinaceus* Mycelia Pilot Double-Blind Placebo-Controlled Study. Front. Aging Neurosci..

[48] Chong P.S., Fung M.-L., Wong K.H., Lim L.W. (2020). Therapeutic Potential of *Hericium erinaceus* for Depressive Disorder. Int. J. Mol. Sci..

[49] Yang C., Mao Z., Wu S., Yin S., Sun Y., Cui D. (2024). Influencing factors, gender differences and the decomposition of inequalities in cognitive function in Chinese older adults: A population-based cohort study. BMC Geriatr..

[50] Kim M., Park J.M. (2017). Factors affecting cognitive function according to gender in community-dwelling elderly individuals. Epidemiol. Health.

[51] Behl T., Kaur D., Sehgal A., Singh S., Sharma N., Zengin G., Andronie-Cioara F.L., Toma M.M., Bungau S., Bumbu A.G. (2021). Role of Monoamine Oxidase Activity in Alzheimer’s Disease: An Insight into the Therapeutic Potential of Inhibitors. Molecules.

[52] Olasehinde T.A., Oyeleye S.I., Ibeji C.U., Oboh G. (2022). Beetroot supplemented diet exhibit anti-amnesic effect via modulation of cholinesterases, purinergic enzymes, monoamine oxidase and attenuation of redox imbalance in the brain of scopolamine treated male rats. Nutr. Neurosci..

[53] Hong R., Li X. (2019). Discovery of monoamine oxidase inhibitors by medicinal chemistry approaches. MedChemComm.

[54] Phan C.W., David P., Naidu M., Wong K.H., Sabaratnam V. (2015). Therapeutic potential of culinary-medicinal mushrooms for the management of neurodegenerative diseases: Diversity, metabolite, and mechanism. Crit. Rev. Biotechnol..

[55] Sepčić K., Sabotič J., Ohm R.O., Drobne D., Jemec Kokalj A. (2019). First evidence of cholinesterase-like activity in Basidiomycota. PLoS ONE.

[56] Lazur J., Hnatyk K., Kała K., Sułkowska-Ziaja K., Muszyńska B. (2023). Discovering the Potential Mechanisms of Medicinal Mushrooms Antidepressant Activity: A Review. Antioxidants.

[57] Sun Y., Cheng L., Zeng X., Zhang X., Liu Y., Wu Z., Weng P. (2021). The intervention of unique plant polysaccharides—Dietary fiber on depression from the gut-brain axis. Int. J. Biol. Macromol..

[58] Cheatham C.L., Nieman D.C., Neilson A.P., Lila M.A. (2022). Enhancing the Cognitive Effects of Flavonoids With Physical Activity: Is There a Case for the Gut Microbiome?. Front. Neurosci..

[59] Whyte A.R., Cheng N., Butler L.T., Lamport D.J., Williams C.M. (2019). Flavonoid-Rich Mixed Berries Maintain and Improve Cognitive Function Over a 6 h Period in Young Healthy Adults. Nutrients.

[60] Cheatham C.L., Canipe L.G., Millsap G., Stegall J.M., Chai S.C., Sheppard K.W., Lila M.A. (2023). Six-month intervention with wild blueberries improved speed of processing in mild cognitive decline: A double-blind, placebo-controlled, randomized clinical trial. Nutr. Neurosci..

[61] Alharbi M.H., Lamport D.J., Dodd G.F., Saunders C., Harkness L., Butler L.T., Spencer J.P. (2016). Flavonoid-rich orange juice is associated with acute improvements in cognitive function in healthy middle-aged males. Eur. J. Nutr..

